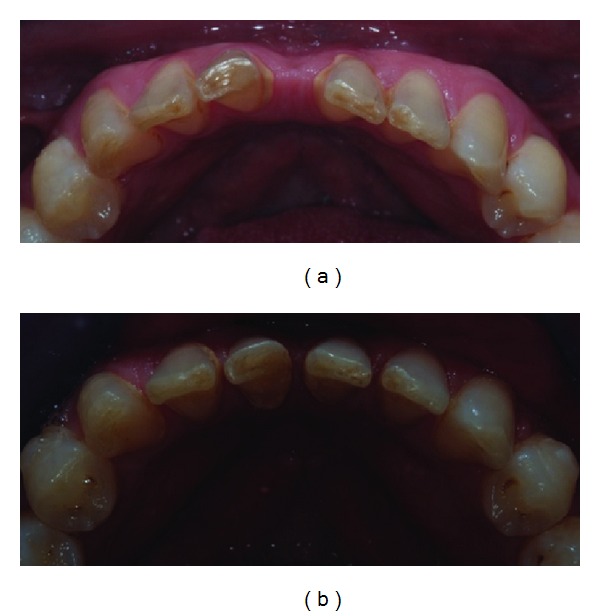# The Anterior Ratio: The Missing Link between Orthodontics and Aesthetic Dentistry

**DOI:** 10.1155/2013/470637

**Published:** 2013-08-19

**Authors:** Raman Aulakh

**Affiliations:** ^1^Aesthetic Dentistry, Kings College, King's College London Dental Institute, Guy's Campus, Floor 18, Tower Wing, St. Thomas' Street, London SE1 9RT, UK; ^2^The Ashcroft Clinic, 2 Ashcroft Drive, Denham, Middlesex UB95JF, UK

## Abstract

There is an increase in the number of dentists using orthodontic treatment for anterior tooth alignment. This is either combined with additive bonding or interproximal reduction; knowing “when to” and “how much” requires an understanding of the anterior ratio. This case report explains how to use the anterior ratio to help provide optimal aesthetics and function for the ortho-restorative patient. The anterior ratio is an important diagnostic tool required for both disciplines of orthodontics and restorative dentistry which is often overlooked. The case report demonstrates the use of the Invisalign orthodontic appliance and the corresponding ClinCheck software to help achieve the proposed treatment goals.

## 1. Introduction

For all orthodontic-restorative cases, the use of the Bolton analysis is an invaluable diagnostic tool which helps to mathematically define a tooth size discrepancy (TSD). A tooth-size discrepancy (TSD) is defined as a disproportion among the sizes of individual teeth. If a tooth size discrepancy does exist with the anterior teeth and is corrected to ideal proportions, the aesthetic smile can be significantly improved [1]. Additionally, correcting a significant TSD can allow for ideal tooth alignment and occlusion in relation to overjet and overbite [2].

**Figure 1:**
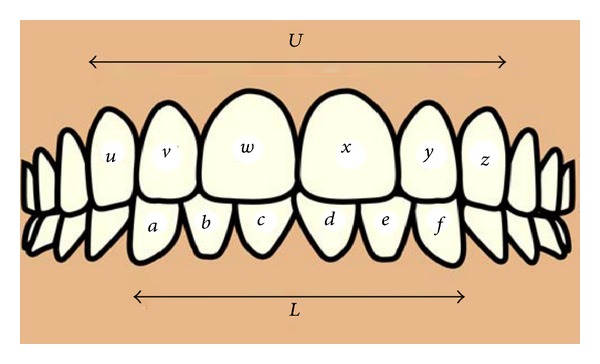
Showing anterior teeth and values required to calculate anterior ratio.

In 1958, Bolton [3] developed a method for estimating a TSD by measuring the summed mesiodistal widths of the mandibular to the maxillary anterior teeth from canine to canine; this was defined as the anterior ratio. The ideal value for the anterior ratio is 0.772 or 77.2% when described as a percentage. This is illustrated in (Figure 1).

The anterior ratio is calculated by the following equation:

(1) $$\begin{array}{c} \text{anterior}\;\text{ratio}=\frac{L}{U}\times 100=77.2\%\pm 1.65\;\left(\text{S}.\text{D}\right), \end{array}$$

where *L* is the sum of the mesiodistal widths of the lower anterior six teeth,

(2) $$\begin{array}{c} L=\sum \left(a+b+c+d+e+f\right), \end{array}$$

where *U* is the sum of the mesiodistal widths of the upper anterior six teeth:

(3) $$\begin{array}{c} U=\sum \left(u+v+w+x+y+z\right). \end{array}$$

Bolton demonstrated that the deviation from the ideal value of any measured ratio would indicate the size of the discrepancy. Therefore as a general rule, an increased anterior ratio value could be due to small upper teeth or large lower teeth or a combination of the two factors. And a decreased anterior ratio could be due to large upper teeth or small lower teeth or a combination of the two factors.

The solution to correcting the anterior ratio is by making an adjustment in the upper arch or in the lower arch or in both arches. The mesiodistal widths of teeth can be increased with composites or indirect restorations and decreased by interproximal reduction and tooth recontouring.

The clinical application of the anterior ratio requires this value to be expressed in actual millimetres required for correction of the discrepancy. Othman and Harradine [4] recommended a threshold of 2 millimetres discrepancy to be of clinical significance. 

The orthodontic appliance Invisalign (Align Technology, Santa Clara, CA, USA) operates with sophisticated digital study model and treatment planning software called ClinCheck. The software makes it easier to manipulate factors such as tooth proportions to diagnostically evaluate different treatment outcomes prior to commencing treatment. This also allows for excellent communication between clinicians for interdisciplinary treatment planning which can then be relayed to the patient.

Krieger et al. [5] have demonstrated that with correct treatment planning, Invisalign treatments in the anterior region do achieve the predicted tooth alignment as demonstrated by the ClinCheck software.

## 2. Case Report

The following example describes a pre-restorative orthodontic case using Invisalign for a Caucasian male aged 38 presenting with a tooth size discrepancy. The anterior ratio was corrected in the lower arch by increasing the mesiodistal widths of the lower incisors by a combination of orthodontics and restorative dentistry.

**Figure 2:**
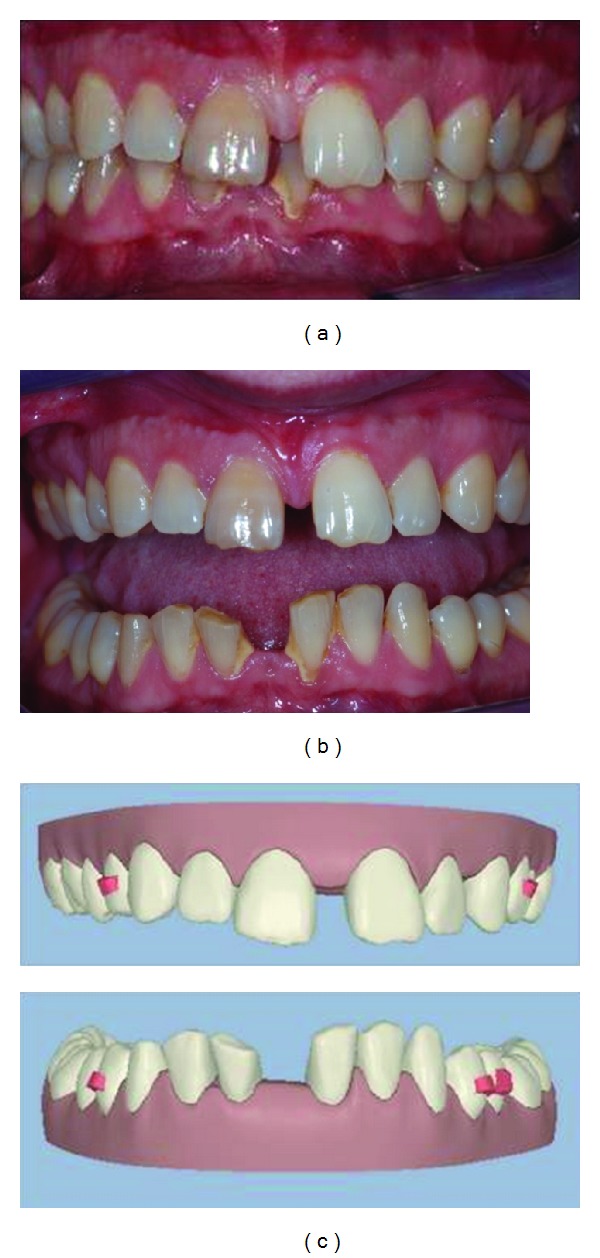


The patient initially presented with a deep bite, class II subdivision and large central diastemas in both arches. The lower incisors were also worn at the incisal level and small in their mesiodistal width as shown in (Figures 2(a) and 2(b)). This initial photo is also seen as a digital study model using ClinCheck software as shown in (Figure 2(c)).

**Figure 3:**
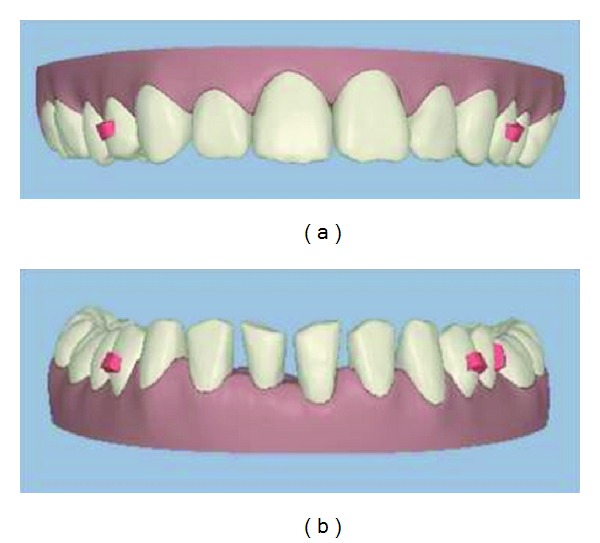


The ClinCheck software allows the clinician to move the teeth into the proposed new position like a virtual digital Kesling setup. In this case the proposed position (Figures 3(a) and 3(b)) was to move the upper incisors together and close the diastema. However, the lower incisors would be moved to leave evenly measured spacing between the lower incisor teeth of 1 mm.

**Figure 4:**
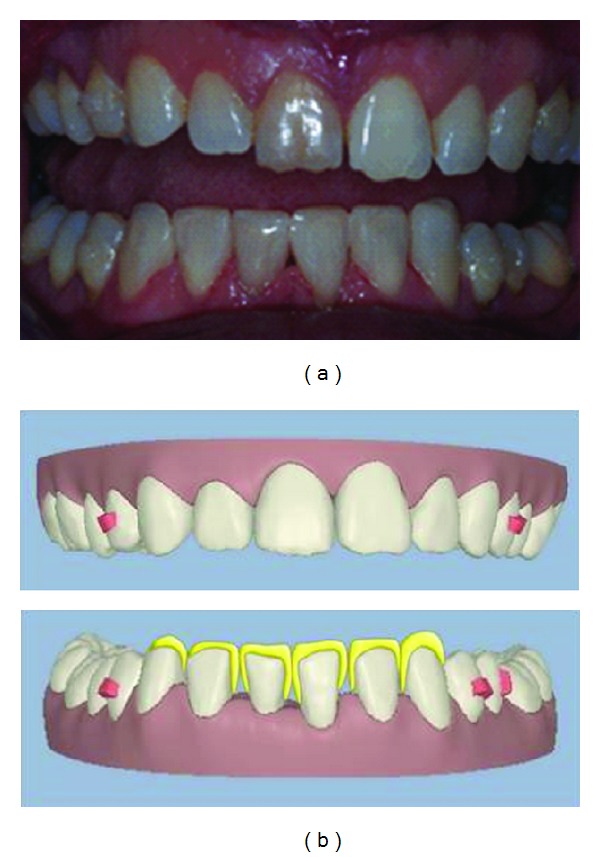


This pre-restorative position would allow the restorative dentist to have space to build the teeth up with composite at both the mesiodistal and incisal level with minimal preparation bonding. This is shown in the final photo in (Figure 4(a)) and also digitally in (Figure 4(b)). The yellow colour represents the additive composite bonding which was performed to achieve the end result after pre-restorative orthodontics. *So why did we build up the lower incisors with composite and close the spaces in the uppers?*

**Figure 5:**
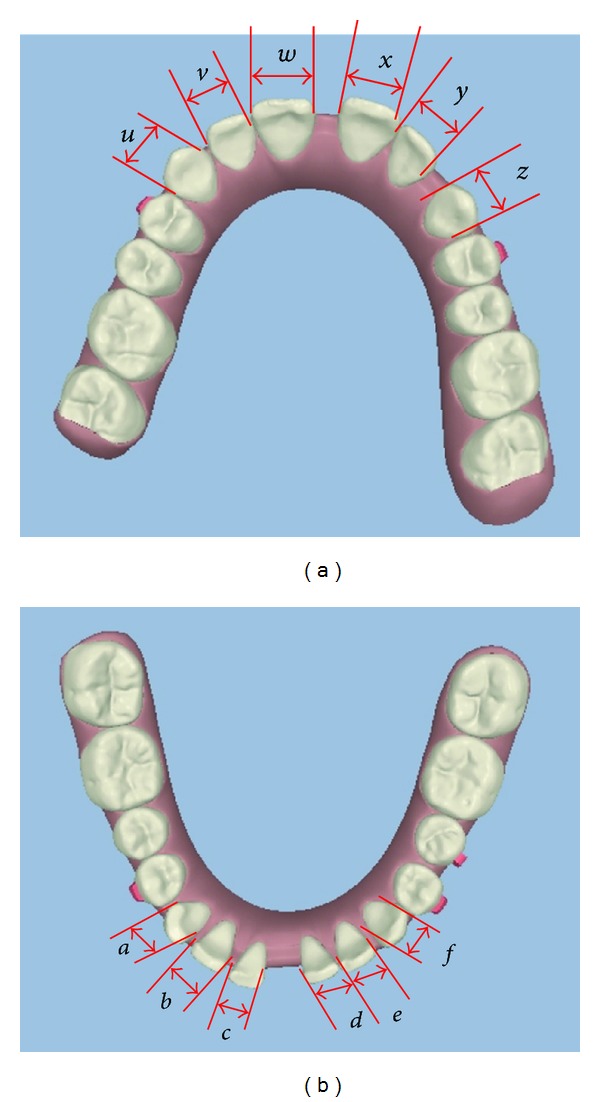


To answer this, we need to calculate the anterior ratio for this case which is described in detail below. (Figures 5(a) and 5(b)) show occlusal views of the patient's original occlusion in digital form using the ClinCheck software with annotation of the measurements required for calculation.

*anterior ratio calculation:*

(4) ∑(a+b+c+d+e+f)∑(u+v+w+x+y+z)×100  =Ideal  77.2%±1.65  (s.d),∑(5+5+4.5+4.5+5+5.5)∑(7+6.5+8.5+8.5+6.5+7)×100=67.0%.

The anterior ratio value for this patient was decreased at 67.0%. This can be clearly recognized clinically by the small lower incisors and canines. Therefore an addition of 5 mm of tooth size would be required in the lower arch to correct the anterior ratio closer to the ideal value. *So what would have happened if we closed all the spaces in both arches and accepted the tooth size discrepancy?*

**Figure 6:**
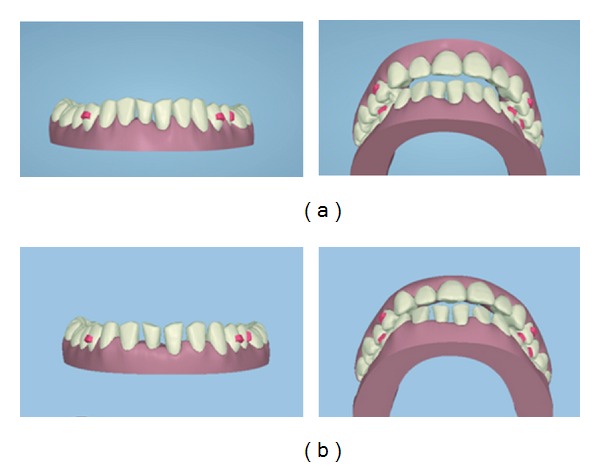
ClinCheck views demonstrating the different proposed treatment plans. (a) Treatment plan no. 1: close all spaces in lower anterior region and accept the TSD tooth size discrepancy. (b) Treatment plan no. 2: evenly distribute spaces between lower incisors to correct the TSD tooth size discrepancy.

This was considered as one of the two treatment options and presented to the patient using the ClinCheck software. Both of the possible solutions are illustrated in (Figures 6(a) and 6(b)) which were considered at the treatment planning stage.

The ClinCheck shows the closure of all spaces; however the overjet has increased due to the reduction of the arch width in the lower arch. There is also a loss of transverse arch coordination at the canine and premolar region due to lower arch constriction from space closure. This will affect the canine coupling and will be detrimental to occlusal function. The TSD has not been corrected; hence the occlusion cannot be improved (see Figure 6(a)). 

The ClinCheck shows even distribution of spaces in-between the lower incisors to aid in correction of the TSD. The pre-restorative orthodontics allows for the incisors to be built up to the correct mesiodistal widths with composite restorations. The correction of the TSD helps to correct the overjet and improve arch coordination with the adjunct of minimally invasive dentistry (see Figure 6(b)).

## 3. Treatment Overview

The duration of the orthodontic treatment was under 10 months in total. The patient was seen for orthodontic appointments on an 8-week basis to check the fit of appliance and supervise treatment progress. A total of 20 aligners were required, with aligners to be worn sequentially for 2 weeks at a time for a minimum of 20 hours a day to achieve the desired movement.

After orthodontic treatment was complete, the lower incisor position can be compared in the pre- and post-treatment photos in (Figures 7(a) and 7(b)). The lower teeth required minimal preparation and only additive bonding with composite to restore them to the correct dimensions. The end position of the lower incisors allowed for adequate space and clearance to build up the incisors without increasing the occlusal vertical dimension. 

## 4. Conclusion

The principle aim of the orthodontic treatment for this case was to assist in space closure in the upper arch and to correct the tooth size discrepancy (TSD) in the lower arch. The buccal relationship and class II subdivision was maintained, however, the anterior occlusion and tooth wear was improved by correcting the deep bite and reducing the overjet. Additionally pre-restorative orthodontics facilitated the restoration of the worn lower anterior incisors.

The orthodontic treatment was performed using Invisalign in both arches. The ClinCheck software provided an excellent tool for interdisciplinary treatment planning and allowed for easy discussion between the orthodontist, restorative dentist, and patient. This case could have been treated with fixed appliance therapy. However, the patient's desire for a highly aesthetic removable brace requiring fewer reviews during treatment was advantageous with the Invisalign appliance.

Understanding and using the anterior ratio as a diagnostic tool can help to solve many orthodontic and aesthetic problems in a systematic and predictable manner. An interdisciplinary approach was used to provide the patient with an optimal aesthetic result while providing minimally invasive treatment. The use of only an orthodontic or restorative approach would not have produced a satisfactory result for this case.

## Figures and Tables

**Figure 7:**